# Enteric Fever and Dysentery in the Army

**Published:** 1903-08-22

**Authors:** 


### Enteric Fever and Dysentery in the Army.

### REPORT OF THE COMMISSION IN SOUTH AFRICA.

The recently issued report of the Commission appointed to inquire into the Nature, Pathology, Causation and Prevention of Dysentery and its Relationship to Enteric Fever, is of interest from many points of view. Although the members of the commission are not quite in agreement as to the exact course to be taken in the future to secure the better safety for soldiers in the field from the diseases to which experience has shown them to be specially liable, yet they are all agreed that something ought to be done with this end in view. The commission, which consisted of Colonel Notter, R.A.M.C, Lieutenant-Colonel Bruce, R.A.M.C., and Professor Simpson, M.D., F.R.C.P., met in Capetown in September, 1900, and forthwith commenced their inquiries. The work was divided into two parts, viz, (1) Laboratory work; (2) inspection of camps, water supply, etc. Lieutenant-Colonel Bruce undertook the first part as his share of the work of the commission, and Colonel Notter and Professor Simpson proceeded to the second part.

The report is divided into three parts, the first of which relates to the bacteriology and pathology of South African dysentery and its relation to enteric fever. The second and third parts deal with the sanitary or health aspects of the campaign, and the physical conditions giving rise to dysentery and enteric fever. The relationship between the two diseases is here recognised to lie in the circumstance that the media by which the causal agents gain an entrance into the body are the same, and that excremental filth plays an important part in the causation and spread of both diseases. Although the conditions favourable for the dissemination of the two illnesses are thus far similar, yet there are other factors which favour dysentery especially; these are decomposition in food, putridity and suspended matter in water, together with chills.

The laboratory work on which the first part of the report is based was carried out in Pretoria, and occupied three months. The short time available and the fact that South African dysentery is not often fatal, so that morbid material was not by any means plentiful, conspired to place limitations on the scope of the inquiry. However, Lieut.-Col. Bruce's conclusions are both interesting and important; they are as follow:—

1. Dysentery in South Africa is not caused by amœbæ, as there is some reason to believe is the case with the dysentery of certain other countries.

2. That the organs in dysentery are absolutely sterile. It is a local disease attacking the mucous and sub-mucous coats of the large intestine, and, unlike enteric fever, the causal agent confines itself to the intestines.

3. That in the large intestines no particular species of micro-organism stands out prominently as in the case of cholera, so that it is impossible in the present stage of investigation to say that any special bacterium plays a prominent part in the causation of dysentery.

4. That there is not sufficient evidence in this work to bring forward the theory that some of the normal inhabitants of the intestines belonging to the coli group take on a pathogenic power.

5. That there is no connection between dysentery and enteric fever. Eberth's bacillus is not found in the organs or intestines of dysentery.

6. That there is a certain amount of evidence to show that so-called cases of dysentery following enteric fever are relapses of enteric, where the disease has attacked the big intestine.

In the second part of the report Dr. Simpson first considers the prevalence of dysentery and enteric fever among the civil population in Cape Colony and Natal and then deals with their occurrence in the Army. It appears that in these countries there were few places that were quite free from either dysentery or enteric fever before the war broke out. This wide prevalence is to be explained in part by the fact that a soil once infected in South Africa retains its infection for a considerable period, while there is an insufficient protection of the water supplies, and a general absence of a proper and sanitary disposal of the excreta and slop water. Moreover, the habits of the natives are filthy and highly conducive to the existence of intestinal diseases. The towns are mostly undrained, and the system of removal of excreta is generally of a very primitive character. Under such circumstances as these it is not surprising that enteric fever and dysentery are rife in South Africa; moreover, the natives suffer from these disorders, and are potent factors in spreading infection.

With regard to enteric fever and dysentery in the Army in South Africa, Dr. Simpson notes the comparative absence of the fatal dysentery which usually attacks armies in the field, and this he attributes to the excellent food arrangements in this campaign. By the introduction of preserved meat in tins, the soldier obtains his rations fresh and already thoroughly cooked, so that the dangers incurred by eating badly preserved rations have now been avoided. During the late war the most frequent cause for the prevalence of dysentery was the drinking of much-polluted surface waters. In many of the rivers the water contained so much suspended matter as to be quite opaque, and the drinking of it by the soldiers was very generally followed by diarrhœa, which in some cases developed subsequently into muco-enteritis.

With regard to the supposed influence of dust storms on the prevalence of enteric fever, Dr. Simpson considers that they have little to do with the causation.

Dr. Simpson concluded that contaminated water was the main vehicle of infection, while flies might play a part by passing to and fro between the latrines and the mess tents.

He sums up the conditions giving rise to the insanitary state of large camps generally under four headings:—

1. Surface supplies of water and their liability to pollution by dead animals or excremental matter, or surface drainage, or all combined.

2. The imperfect and ill-considered disposal of carcases of horses and oxen.

3. Defective covering of latrines, proximity of litter, manure, and refuse heaps, and fouling of the ground with urine and slop water, causing risk of infection of food from flies and from the possible contamination of boots, clothes, utensils, and hands.

4. The absence of organised establishments for the cleansing of camps that have been occupied; the absence also of arrangements for the distribution and location of regiments on clean ground, and the consequent occupation of the same camps successively by different regiments.

Colonel Notter's conclusions coincide pretty closely with those of Dr. Simpson except that the former lays more stress on the evil influence of the close aggregation of men and animals in small encampments, and the part played by flies in carrying infection.

